# Macrophage membrane-functionalized biomimetic Yiqi Huoxue formula nanoparticles improve atherosclerosis by regulating smooth muscle cell phenotypic transition via the KLF4/NF-κB pathway

**DOI:** 10.1186/s13020-026-01477-y

**Published:** 2026-07-27

**Authors:** Yanqing Li, Hongyuan Wang, Yi Guan, Jinli Zhuang, Xiaohong Yang, Yanting Chen, Shengyu Cun, Jianfeng Li, Ya Qin, Quan Xie

**Affiliations:** 1 Department of TCM & Western Medicine, Affiliated Reproductive Maternity and Child Hospital of Chengdu University of Traditional Chinese Medicine, Chengdu, China; 2 Chengdu University of Traditional Chinese Medicine, Chengdu, China; 3 Hospital of Chengdu University of Traditional Chinese Medicine, Chengdu, China

**Keywords:** Atherosclerosis, YQHXF, Nanoparticles, Foam cells, KLF4, NF-κB

## Abstract

This study aimed to analyze the active ingredients of the compound preparation of Yiqi Huoxue (YQHX) and evaluate the therapeutic effect of its nanoparticles (MM/YQHXF-NPs) on atherosclerosis (AS). First, the active ingredient in the YQHX formulation was identified by LC–MS analysis. Subsequently, transmission electron microscopy (TEM) tests particle size. Mapping tests nanoparticle surface elements. Dynamic light scattering (DLS) tests nanoparticle size and distribution. ZETA tests nanoparticle surface potential. HPLC tests drug release. The results showed that these nanoparticles were spherical, approximately 100 nm in size, and had good dispersion. The P element content of MM/YQHXF-NPs increased after cell membrane coating, and their hydrodynamic size also increased accordingly, but the Polymer dispersity index (PDI) value was low, indicating good monodispersity. In addition, the nanoparticle surface had a weak negative charge, the encapsulation efficiency of YQHXF was 59.4%, and the drug loading rate was 5.61%. In cell-based experiments, MM/YQHXF-NPs showed no cytotoxicity towards A7r5 cells at a concentration of 150 μg/mL. The study found that ox-LDL-induced A7r5 cell-to-foam cell transformation was significantly inhibited. Oil Red O staining revealed that MM/YQHXF-NPs reduced lipid accumulation. In addition, YQHXF and its active component, salvianolic acid B, can inhibit the foam cell formation of A7r5 cells. Furthermore, MM/YQHXF-NPs modulated the phenotype of smooth muscle cells, inhibiting the expression of genes such as *Myh9*, *Icam-1*, *Vcam-1*, *Tnfrsf11b*, *Cd68*, *Lgals3*, and *Abca1*, while promoting the expression of *Myh11* and *Smtn*. Mechanistic studies revealed that MM/YQHXF-NPs exerted their effects by inhibiting the Krüppel-like factor 4 (KLF4) and NF-κB signaling pathways. In a high-fat diet, *ApoE*^−/−^ mice model of AS, MM/YQHXF-NPs demonstrated significant therapeutic efficacy. H&E and Oil Red O staining revealed that MM/YQHXF-NPs mitigated pathological changes, reduced plaque size, and lowered serum TC, TG, LDL, and HDL levels. They also stabilized atherosclerotic plaques by increasing fiber area and promoting SM22α and SM-MHC expression. Consistent with the results from cell-based experiments, MM/YQHXF-NPs effectively inhibited the transformation of arterial smooth muscle cells (SMCs) into foam cells in vivo and suppressed the activation of KLF4 and NF-κB signaling pathways. In summary, MM/YQHXF-NPs can effectively prevent the transformation of SMCs into foam cells by inhibiting the KLF4 and NF-κB signaling pathways, thereby alleviating AS. These results provide a theoretical basis for MM/YQHXF-NPs as a potential therapeutic drug for AS.

**Supplementary Information:**

The online version contains supplementary material available at 10.1186/s13020-026-01477-y.

## Introduction

Atherosclerosis (AS) is the most important pathological basis of cardiovascular disease and can cause ischemic cardiovascular and cerebrovascular diseases, such as cerebrovascular disease, coronary heart disease, and peripheral arterial disease [1, 2]. AS is a chronic inflammatory disease of the arterial wall, the main cause of which is lipid metabolism disorder and inflammatory response disorder [3, 4]. The accumulation of lipids in the arterial wall and the maladaptive inflammatory response can lead to arterial thickening and hardening [5]. The thickening and hardening of the blood vessel wall can narrow the arterial lumen, making it difficult for blood to pass through, thereby causing various ischemic cardiovascular and cerebrovascular diseases. Additionally, rupture of AS plaques can lead to intravascular thrombosis. Acute thrombosis can block the flow of blood in the artery, leading to fatal clinical events such as myocardial infarction (MI) and heart failure, as well as ischemic stroke in the cerebral arteries [6]. Therefore, it is urgent to explore the pathogenesis and treatment of AS.

Foam cell formation is an early event in the development of AS [7]. Foam cells are macrophages or smooth muscle cells (SMCs) that phagocytize large amounts of fat [8]. Vascular smooth muscle cells (VSMCs) are the most numerous cells in the arterial wall. In healthy arteries, they are contractile and found in the medial layer [9]. However, various stimuli lead to the loss of this contractile phenotype, migration to the intima, and further phenotypic conversion [10]. In a process called diffuse intimal thickening (DIT), intimal VSMCs proliferate, degrade the collagen extracellular matrix (ECM), and secrete proteoglycans, which bind to apolipoprotein B (apoB)-containing lipoproteins in the subendothelial space [11]. Within the intima, lipid pools undergo oxidation and other modifications [12]. This leads to the recruitment of circulating macrophages or the proliferation of tissue-resident macrophages, an inflammatory response, and the phagocytosis of lipids [12]. This transformation into so-called foam cells is a key component of the early pathological intimal thickening (PIT) phase of AS [13]. Following apoptosis and subsequent necrosis, foam cells release further inflammatory molecules, ultimately leading to the formation of a fibrous cap [8].

Previous studies have confirmed that Krüppel-like factor 4 (KLF4) is an important therapeutic target for AS [14, 15]. As a multifunctional transcription factor, the core pathophysiological mechanisms of KLF4 primarily involve vascular endothelial cells (ECs) and VSMCs; the activation and inhibition of its molecular signaling pathways directly regulate vascular inflammation, phenotypic switching, and plaque stability [16, 17]. Specific knockout of KLF4 in VSMCs reduces atherosclerotic lesions and increases fibrous cap thickness, demonstrating that KLF4 deficiency inhibits the transformation of VSMCs into macrophage-like and mesenchymal-like VSMCs and promotes plaque stabilization [14, 15]. The NF-κB signaling pathway is closely associated with AS, a chronic inflammatory disease associated with an immune response at the onset and throughout its course [18, 19]. Studies have reported that reducing KLF4 expression can inhibit NF-κB p65 activation, thereby protecting against cardiovascular damage [20, 21]. Therefore, the KLF4/NF-κB signaling pathway is crucial for the transformation of SMCs into macrophages in AS.

Traditional Chinese medicine (TCM) has a long history and unique advantages in preventing and treating AS. Many TCM herbs and their active ingredients exhibit multi-target and multi-pathway effects, including regulating blood lipids, providing anti-inflammatory and antioxidant benefits, and inhibiting the proliferation of vascular smooth muscle cells, thereby exerting anti-atherosclerotic effects [22, 23]. For example, Traditional Chinese herbal medicines such as Ren Shen [24], Dan Shen [25], Chuan Xiong [26], and San Qi [27] have been widely used in the treatment of AS with considerable success. In TCM, Yiqi Huoxue is a classic theory used to treat AS, as well as cardiovascular and cerebrovascular diseases [28, 29]. The Yiqi Huoxue formula (YQHXF), comprising Hong Shen, Cang Zhu, Dan Shen, Chuan Xiong, and San Qi, is a commonly used remedy in the clinical treatment of AS. YQHXF has been demonstrated in both clinical and basic research to exhibit significant therapeutic efficacy against cardiovascular diseases, including AS [28, 30]. Salvianolic acid B, an active constituent of Dan Shen, possesses established anti-atherosclerotic properties and exerts an inhibitory effect on the NF-κB signaling pathway [31, 32]. However, the active ingredients of TCM often have problems such as low solubility, poor bioavailability, and short half-life in vivo, which limit their clinical application [33]. With the cross-integration and rapid development of materials science, nanotechnology, and biomedical engineering, a new research field has emerged—nanomedicine, which has brought new breakthroughs in the diagnosis and treatment of various diseases [34, 35]. In general, the design of nanomaterial delivery systems can solubilize drugs, increase drug half-life, improve drug distribution in the body, enhance targeting, and reduce toxic side effects. It has great application potential in the field of disease diagnosis and treatment [36]. However, there are few reports on the research of YQHXF nanoparticles in AS.

Therefore, this study seeks to elucidate the therapeutic effects of YQHXF nanoparticles on AS, with a particular emphasis on the mechanistic role of KLF4/NF-κB in this pathological condition. Additionally, we aim to explore the influence of YQHXF on the transdifferentiation of vascular SMCs into foam cells via the KLF4/NF-κB pathway. The findings from this research are anticipated to contribute significantly to the theoretical and experimental foundations necessary for the development of novel therapeutic strategies and pharmacological interventions. Ultimately, this study aspires to propose more effective strategies and methodologies for enhancing the prognosis and quality of life for patients afflicted with AS.

## Materials and methods

### Identification of the material composition of YQHXF

YQHXF is composed of Hong Shen (6 g), Cang Zhu (3 g), Dan Shen (3 g), Chuan Xiong (3 g), and San Qi (3 g). The pharmacy of Chengdu University of Traditional Chinese Medicine provided the extract of YQHXF. In this project, the target compounds were separated chromatographically using a Vanquish (Thermo Fisher Scientific) ultra-high performance liquid chromatograph (UPLC) on a Phenomenex Kinetex C18 (2.1 mm × 100 mm, 2.6 μm) column. Phase A consisted of an aqueous phase containing 0.01% acetic acid, and phase B consisted of isopropanol: acetonitrile (1:1, v/v). The sample tray temperature was 4 °C, and the injection volume was 2 μL. An Orbitrap Exploris 120 mass spectrometer, controlled by Xcalibur software (version 4.4, Thermo), acquired both primary and secondary mass spectrometric data. The detailed parameters of the DDA method are as follows: sheath gas flow rate: 50 Arb, aux gas flow rate: 15 Arb, capillary temperature: 320 °C, full ms resolution: 60,000, MS/MS resolution: 15,000, collision energy: SNCE 20/30/40, spray voltage: 3.8 kV (positive) or − 3.4 kV (negative).

### Construction and identification of MM/YQHXF-NPs

A 100 mg sample of PLGA-COOH was dissolved in 3 mL of chloroform and sonicated. Separately, 10 mg of DiD was dissolved in 1 mL of chloroform and then added to the chloroform containing the PLGA-COOH. The drug was added and thoroughly mixed. The resulting mixture was sonicated on ice for a total duration of 30 s, with a 2-s interval between each 3-s application of 120 W power. The primary emulsion that was formed was then dispersed into 10 mL of a 2% (w/v) aqueous solution of polyvinyl alcohol (PVA). This mixture was then sonicated on ice for 10 min, with 2-s intervals between each 3-s application of 120 W power. The sonicated emulsion was transferred to a 50 mL three-necked flask and sonicated at 500 rpm/min for 3 h, which allowed the chloroform to evaporate. Subsequently, the emulsion was washed three times by centrifugation, and the sample was collected and stored at 4 °C until it was ready for use. Finally, macrophage membranes were added and sonicated on ice for 5 min. The probe was then ultrasonicated for 2 min (with 5-s on and 5-s off cycles), and the emulsion was washed by centrifugation. Transmission electron microscopy (TEM) tests particle size. Mapping tests nanoparticle surface elements. Dynamic light scattering (DLS) tests nanoparticle size and distribution. ZETA tests nanoparticle surface potential. The drug release behavior was evaluated, and the concentration of the released components was quantified using HPLC.

### Cell culture

Rat thoracic aorta SMCs A7r5 (Cat. No. GNR7) were provided by the Cell Bank of the Chinese Academy of Sciences. A7r5 cells were subcultured in DMEM medium supplemented with 10% fetal bovine serum (C04001-500, Vivacell) and 100 U/mL of penicillin–streptomycin. Cells were incubated at 37 °C in a 5% carbon dioxide (CO_2_) incubator. When cells reached logarithmic phase, the old culture medium was removed, cells were rinsed with phosphate-buffered saline, and digested with 1 mL of trypsin. Subculture continued until the intercellular spaces increased and the cells became rounded.

### CCK-8 assay

A7r5 cells in the logarithmic growth phase were subjected to washing with phosphate-buffered saline (PBS) and subsequently digested with trypsin for harvesting. The cells were then centrifuged at 250*g* for 5 min, after which the supernatant was removed by aspiration. An appropriate volume of culture medium was added to form a cell suspension, and the cell density was adjusted to 5 × 10^4^ cells/mL. A volume of 100 μL per well was dispensed into a 96-well plate, with the peripheral wells filled with sterile PBS, and incubated at 37 °C in a 5% CO_2_ atmosphere. Following 24 h of drug exposure, the supernatant was aspirated and discarded. The Cell Counting Kit-8 (CCK-8, BS350B, Biosharp) reagent was diluted at a ratio of 1:10 in serum-free culture medium, and 110 μL of the diluted CCK-8 working solution was added to each well. The plate was gently agitated several times and incubated again at 37 °C in a 5% CO_2_ environment for an additional 2 h. Absorbance was measured at 450 nm using a microplate reader to assess cell viability.

### Oil Red O staining

After the cell experiment, the A7r5 cells were washed twice with PBS and fixed with Oil Red O fixative for 20 min. The fixative was discarded, and the cells were washed twice with distilled water. The cells were then immersed in 60% isopropanol for 20–30 s. The 60% isopropanol was discarded, and a freshly prepared Oil Red O staining solution (G1262, Solarbio) was added for 15 min. The staining solution was discarded, and the cells were rinsed with 60% isopropanol for 20–30 s until the interstitial tissue was clear. The cells were washed with water 2–5 times, discarded, covered with distilled water, and observed and photographed under a microscope.

After the animal experiment, the aorta was removed and fixed with fixative for 24 h. The aorta was first immersed in isopropyl alcohol and then stained in Oil Red O solution (YO7512, bomei) at 37 °C in the dark. The aorta was then immersed in isopropyl alcohol until the fatty plaques in the lumen turned red and other parts were almost colorless. The blood vessel was then removed, and excess water was removed. The overall plaque formation in the aorta was observed under a microscope. Images were collected, and the gross Oil Red fat area percentage was analyzed using Image-Pro Plus 6.0 software.

### RT-qPCR experiments

Total RNA was extracted from A7r5 cells and mouse arteries using the Trizol Total RNA Extraction Kit (BL1365A, biosharp). The isolated RNA was then reverse transcribed into complementary DNA (cDNA) using the Reverse Transcription Kit (RR047A, TaKaRa). Gene expression analysis was conducted with the SYBR Premix Ex Taq™ II kit (RR820A, Japan). Data were analyzed using the relative quantification method, specifically the ΔΔCt method. For each sample, the mean Ct value was calculated, and relative mRNA expression levels were determined using the 2^−ΔΔCt^ method. The primer sequences employed in this study are presented in Table 1.

**Table 1:** Primers and base sequences used in this research

Species	Gene	Upstream	Downstream
Rat	*β-actin*	gggaaatcgtgcgtgacatt	gcggcagtggccatctc
	*Abca1*	gcgagctgcgttctaacatg	caacgttgtggtggcttcag
	*Cd68*	cctgtgtgtctgaccttgct	aaggatggcagaagagtggc
	*Icam-1*	cggtgctcaggtatccatcc	gttagtctccaaccccaggc
	*Lgals3*	gtgcttatcctggcccaact	aaggggccagtagcaggata
	*Myh9*	aggaagagctggaggaggag	cgcgcattctcgttcttctg
	*Myh11*	aatgtgcacgagctggagaa	cgagcctggagatctcgttc
	*Smtn*	gggcagtatcttcgaccgag	tctcaatcatggccttgcgt
	*Smtn*	gggcagtatcttcgaccgag	tctcaatcatggccttgcgt
	*Vcam-1*	actgtgacctgtcagcgaag	ttagggaccgtgcagttgac
Mouse	*β-actin*	ctacctcatgaagatcctgacc	cacagcttctctttgatgtcac
	*Abca1*	gggtgtctacgtgcaacaga	gcgacagagtagatccaggc
	*Cd68*	cagggaggttgtgacggtac	actcgggctctgatgtaggt
	*Icam-1*	taatgtctccgaggccagga	cgagcttcagaggcaggaaa
	*Lgals3*	tcctggttgaagctgaccac	agttggctgatttcccggag
	*Myh9*	cagcagctgttcaaccacac	catctagcagggccaggatg
	*Myh11*	gcgccttttccgatggattc	gtgtggttgaacagctgctg
	*Smtn*	gggcagtatcttcgaccgag	tctcaatcatggccttgcgt
	*Vcam-1*	ttgacatctcccccggatct	tggatttggccccctcattc

### Western blot assay

A7r5 cells and minced aortic tissue were added to the lysis buffer and homogenized in a grinder. The supernatant was centrifuged, and the total protein concentration was determined using a BCA protein assay kit. The protein sample was mixed with protein loading buffer and heated at 100 °C for 10 min. Proteins were separated by sodium dodecyl sulfate–polyacrylamide gel (SDS-PAGE) electrophoresis. The membranes were transferred to PVDF membranes, blocked for 15 min, and incubated with primary antibodies against KLF4 (A13673, 1:1000, Abclonal), p65 (AF5006, 1:2000, Affinity), p-p65 (AF2006, 1:1000, Affinity), VCAM-1 (DF6082, 1:1000, Affinity), and MCP-1 (507277, 1:500, zenbio) overnight at 4 °C. The following day, secondary antibodies (AS014, 1:10000, Abclonal) were incubated at room temperature, and ECL (Biosharp, BL520B) development was performed. The grayscale values of each protein band were analyzed using ImageJ software. The relative expression levels of the target proteins were calculated using β-actin (AC026, 1:50000, Abclonal) as an internal control.

### Transfection experiments

A7r5 cells in the logarithmic growth phase were seeded at a density of 2 × 10^5^ cells/mL, with 2 mL per well in a 6-well plate, and incubated at 37 °C with 5% CO_2_ for 24 h. Upon cell attachment, two 1.5 mL centrifuge tubes were prepared, each containing 300 μL of Opti-MEM™ I medium (31985070, Gibco™). 15 μg of target plasmid was added to one tube, and 30 μL of Lipo 3000 transfection reagent (L3000001, Thermofisher) was added to the other tube and the mixture was pipetted, mixed, and allowed to stand for 5 min. The contents of each tube were mixed by pipetting and allowed to incubate for 5 min. Subsequently, the plasmid-containing medium was gently combined with the Lipo 3000-containing medium, mixed by pipetting, incubated for an additional 10 min, and the total volume was adjusted to 6 mL. The supernatant from the wells was aspirated, and the transfection complex was added to the cells. The cells were then cultured at 37 °C with 5% CO_2_. After 6 h, the medium was replaced with complete culture medium, and cell culture was continued. The OV-NC and OV-KLF4 plasmids were constructed by Shanghai Genepharma.

### Animal experiments

SPF-grade *ApoE*^−/−^ mice (male, 6–8 weeks old, 18–22 g, 30 mice) and C57BL/6 mice (male, 6–8 weeks old, 18–22 g, 6 mice) were provided by spfbiotech (Beijing) Co., Ltd. (SCXK(Beijing)2024-0001). The experimental animals were housed in a barrier system with a temperature range of 20 °C to 26 °C, a relative humidity of 30% to 70%, and a 12-h day/12-h night light cycle. These mice were divided into the Control group (C57BL/6), Model group (*ApoE*^−/−^), NPs group (*ApoE*^−/−^), MM/YQHXF-NPs group (*ApoE*^−/−^), YQHXF-NPs group (*ApoE*^−/−^), and YQHXF group (*ApoE*^−/−^), with 6 mice in each group. Apart from the control group, which received a standard diet, the experimental groups were administered a high-fat diet for a duration of 12 weeks to establish a high-fat model. Mice in the YQHXF group received oral gavage of 7.02 g/kg/d YQHXF (crude drug equivalent) once a day for 30 consecutive days. The control and model groups received oral gavage of equal amounts of normal saline at the same time points. Mice in the NPS group, YQHXF-NPS group, and MM/YQHXF-NPS group were injected into the tail vein once every 3 days at 30 mg/kg for 30 days. A high-fat diet was maintained during administration, and the control group was fed with ordinary control feed. After the end of the treatment, the mice were anesthetized by intraperitoneal injection of 50 mg/kg of sodium pentobarbital and then killed, and samples were collected for testing. All experiments were approved by the Ethics Committee of West China Hospital Experimental Animal (20250814010).

### H&E staining

Arterial tissue was fixed overnight with paraformaldehyde and sliced into approximately 3 mm sections. After dehydration with graded ethanol, clearing with xylene, wax immersion, and embedding, sections were cut into 4 μm thin sections using a microtome. The wax strips were sectioned and placed in 37 °C warm water to remove the sections and dry them. The sections were then washed with xylene, ethanol, and distilled water, followed by H&E staining (H9627, Sigma Aldrich), mounted with neutral gum, and observed under a microscope for tissue damage.

### Kits and ELISA tests

The plasma of mice in each group was collected and centrifuged at 4 °C and 1500 rpm for 15 min. The supernatant was taken, and the contents of T-CHO (A111-2-1, Nanjing Jiancheng Bioengineering Institute), TG (A110-2-1, Nanjing Jiancheng Bioengineering Institute), Mouse LDL ELISA KIT (C-38097, Zcibio), and Mouse HDL ELISA KIT (ZC-38175, Zcibio) in serum were determined according to the instructions of the kit. The absorbance (OD value) was measured at a wavelength of 450 nm using an enzyme marker to calculate the sample concentration.

### Masson staining

Masson staining (G1006, Servicebio) was used to detect intracoronary plaque fibrosis. Paraffin-embedded arterial tissue was prepared into 4 μm sections. Sections were incubated overnight with potassium dichromate, then heated in a 63 °C oven for 1 h. Sections were then stained with Ponceau fuchsin for 10 min and briefly rinsed with distilled water. Sections were treated with a phosphomolybdic acid solution for 2 min to a few seconds, until the collagen fibers faded. Sections were then stained with aniline blue for approximately 2 min until the collagen fibers were stained. Sections were then dehydrated with graded alcohol, cleared with a clearing agent, and mounted with neutral gum. Observation and analysis were performed under a light microscope.

### Picrosirius Red staining

Arterial paraffin sections were oven-dried at 65 °C for 30 min, dewaxed in xylene for 10 min, washed three times with 100% ethanol (2 times for 3 min each), 95% ethanol (2 times for 3 min each), 80% ethanol (3 min each), and 70% ethanol (3 min each). Sections were then rinsed in tap water and stained with picrosirius red solution (G1472, solarbio) for 1 h. Sections were rinsed in tap water and sequentially treated in 100% ethanol, 95% ethanol, 80% ethanol, 70% ethanol, and xylene for 5 min each. Sections were mounted with resin, dried, and photographed under a microscope. Images were analyzed using Image J software to determine the proportion of fibrotic area relative to the total area, and the results were statistically analyzed.

### Immunofluorescence staining assay

Paraffin blocks of mouse artery tissue were made into sections, and the deparaffinized and hydrated tissue sections were added with an appropriate amount of antigen repair solution (citrate buffer), and thermally repaired for 15 min in a microwave oven. Then the sections were rinsed 3 times with PBS. The sections were sealed with bovine serum (Servicebio, GC305010) for 30 min to minimize non-specific staining. SM-MHC antibody (Abcam, ab234318, 1:200), SM22α antibody (proteintech, 10493-1-AP, 1:300), and KLF4 antibody (proteintech, 11880-1-AP, 1:200), p-p65 antibody (bioss, BS-0982R, 1:100), CD68 antibody (Affinity, DF7518, 1:100) and α-SMA antibody (proteintech, 67735-1-ig, 1:100) were incubated overnight in a wet box at 4 °C. Subsequently, following PBST washes of the sections, diluted fluorescent FITC-conjugated goat anti-rabbit (Servicebio, GB22303, 1:100) was used to label SM22α and SM-MHC. In addition, HRP-labeled goat anti-rabbit IgG was used to label KLF4 and p-p65. Signal amplification was then achieved by incubating with TSA (CY5-Tyramide, Servicebio, G1232, 1:500) in the dark for 10 min at room temperature. The tissue sections were then heat-fixed to expose the antigenic epitopes. Two primary antibodies (CD68 and α-SMA) were added sequentially for overnight incubation. Fluorescently labeled secondary antibodies, FITC-labeled anti-rabbit (Servicebio, GB22303, 1:100) and CY3-labeled anti-mouse (Servicebio, GB21301, 1:200) were then added to detect and label the binding of the primary antibodies. Next, DAPI was added dropwise to the slices and incubated at room temperature for 10 min, and then washed 3 times with PBST. Finally, the slides were mounted with mounting fluid containing an anti-fluorescence quenching agent, and then the images were observed and collected under a fluorescence microscope.

### Statistics and analysis

All data were analyzed utilizing SPSS version 20.0, while statistical visualizations were generated using GraphPad Prism version 8.0. The normality of the data distribution was assessed via the Shapiro–Wilk test. Data conforming to a normal distribution were reported as mean ± standard deviation ($\overline{{\mathrm{x}}}$ ± s). Multiple-group comparisons were performed using one-way ANOVA; the LSD-t test was applied when variances were homogeneous, while Dunnett's t-test was used when variances were heterogeneous. A p-value of less than 0.05 was considered indicative of statistical significance.

## Results

### The analysis of active ingredients of YQHX formula

**Fig. 1:**
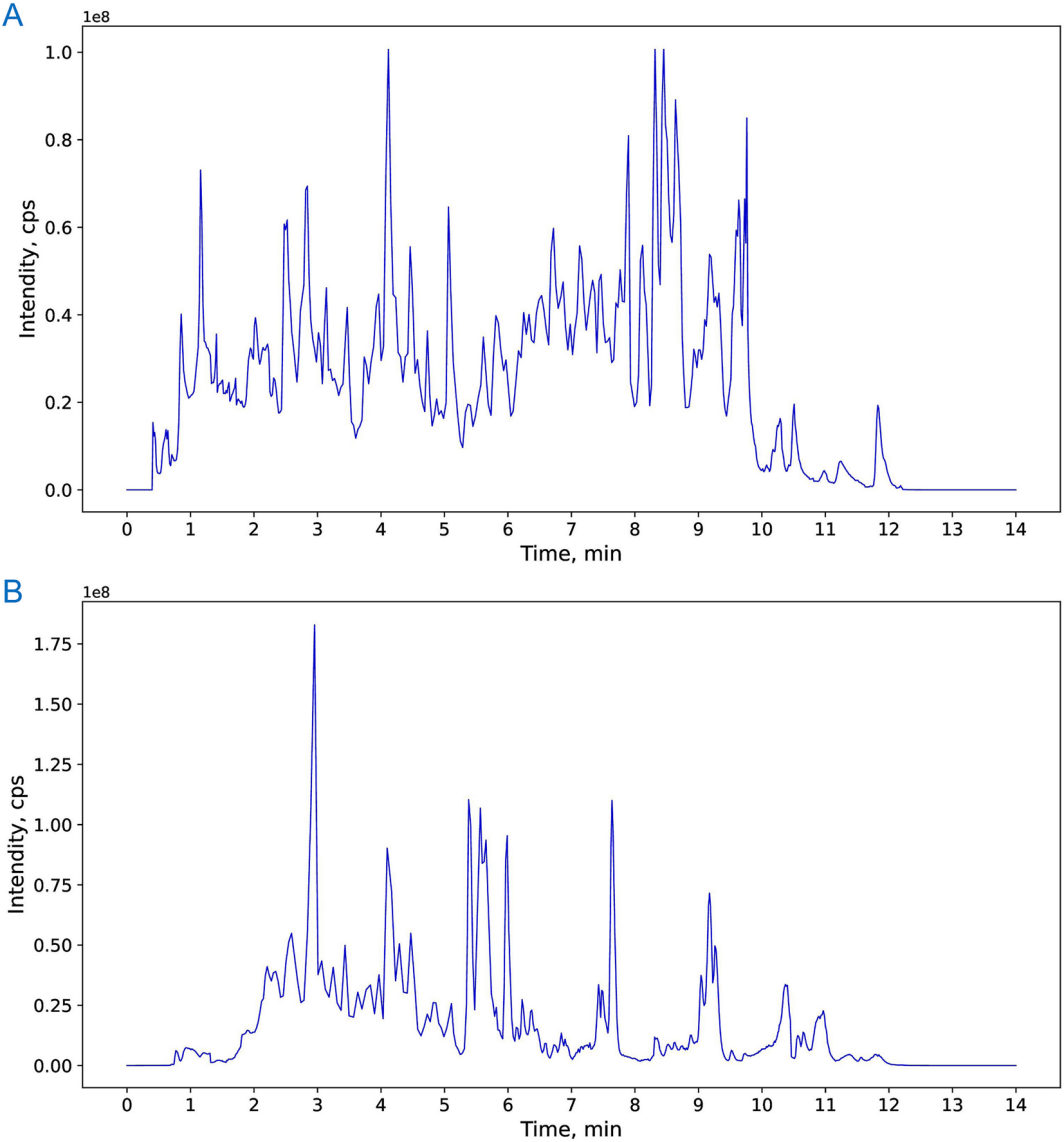
Chromatograms depicting the positive and negative ions of the YQHX formula. **A** Chromatograms representing the positive ions of the YQHX formula. **B** Chromatograms illustrating the negative ions of the YQHX formula

Figure 1 illustrates chromatograms representing the positive (Fig. 1A) and negative (Fig. 1B) ion modes of the YQHX formulation. The raw LC–MS data underwent preprocessing procedures, including peak extraction, noise reduction, deconvolution, and peak alignment, using Xcalibur software. Detailed information concerning the active constituents identified in the chromatograms for both the positive and negative ion modes of the YQHX formulation is presented in Table S1. Table S1 provides a comprehensive chemical analysis of the YQHX formula, identifying Salvianolic acid B, a compound whose distinct anti-atherosclerotic effects have previously been reported [31]. The quantitative analysis of Salvianolic acid B content is presented in the Supporting Information. Furthermore, the content of Salvianolic acid B, as well as the drug loading capacity and encapsulation efficiency, are presented.

### Characteristics of YQHX formula nanoparticles

**Fig. 2:**
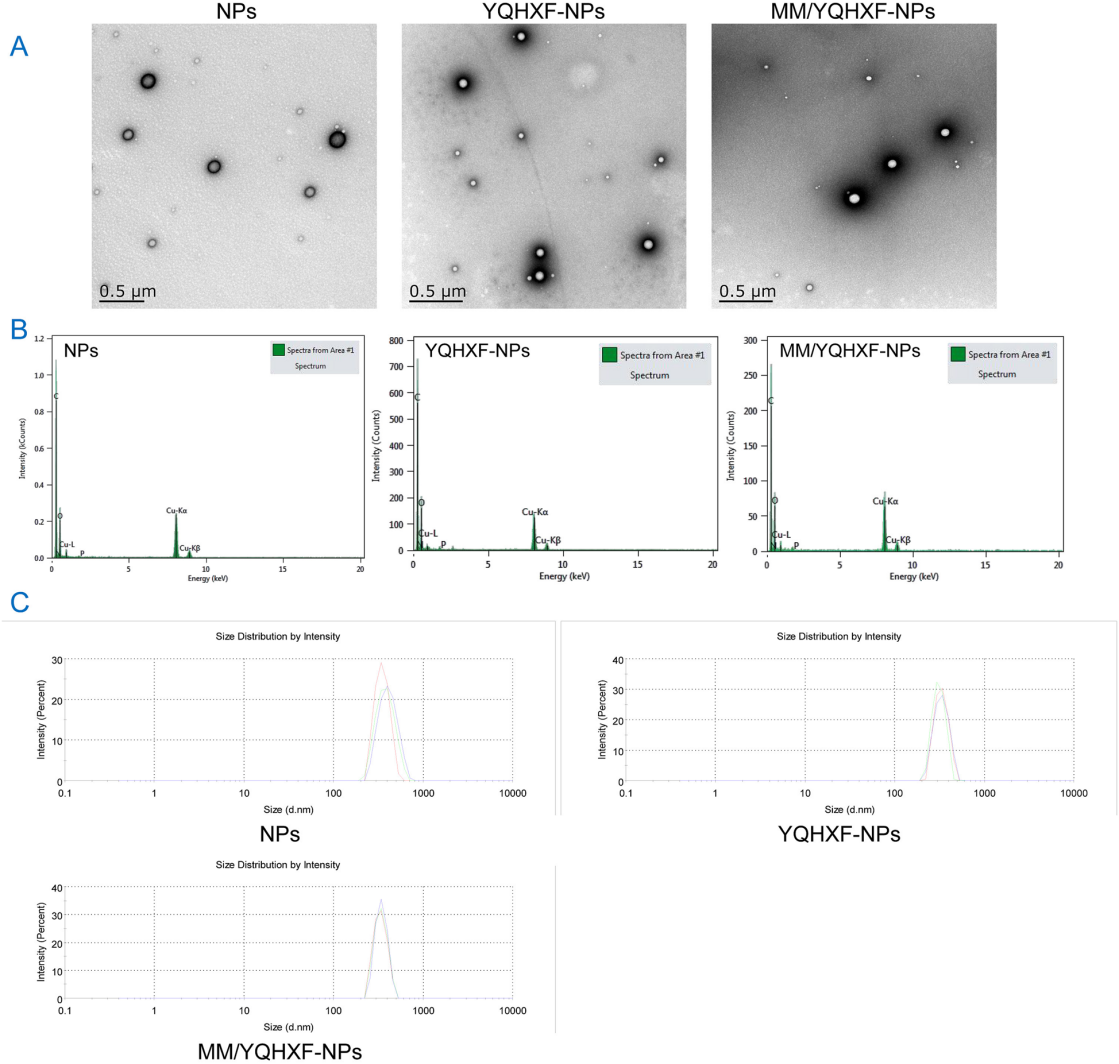
Characterization of macrophage membrane-coated nanomaterials. **A** The image of NPS, YQHXF-NPS, and MM/YQHXF-NPS was detected by TEM. **B** Mapping detection results. **C** A representative image of mapping detection

**Fig. 3:**
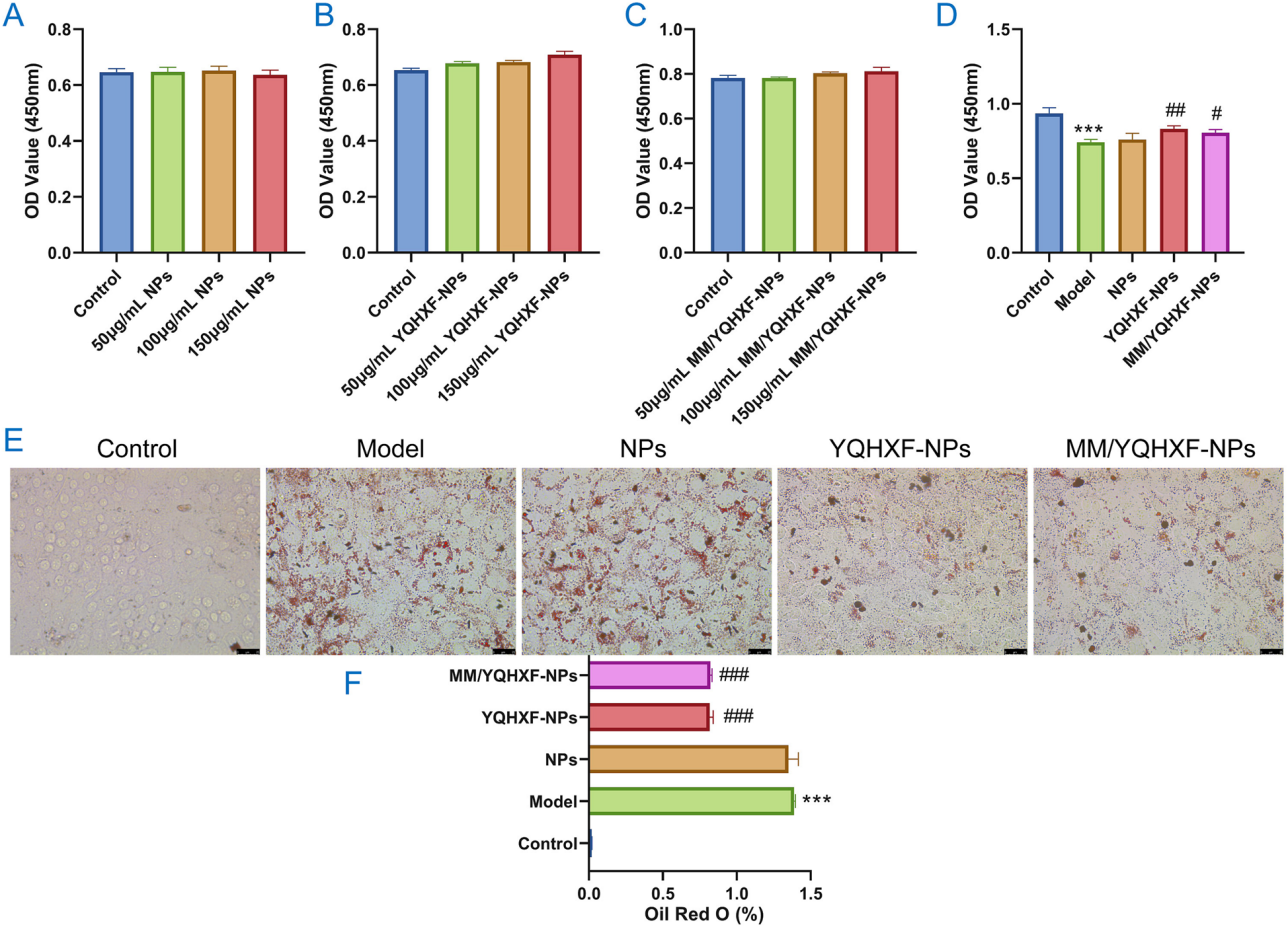
Inhibition of smooth muscle cell-to-foam cell conversion by MM/YQHXF-NPS. **A**–**C** Cytotoxic effects of NPS, YQHXF-NPS, and MM/YQHXF-NPS on A7r5 cells. **D** Effects of 150 μg/mL NPS, YQHXF-NPS, and MM/YQHXF-NPS on the proliferation of foam-forming A7r5 cells. **E**, **F** Representative images of Oil Red O staining and statistical results. Data are presented as mean ± standard deviation, n = 3. Compared with the control group, ***p < 0.001. Compared with the model group, ^#^P < 0.05, ^##^P < 0.01, and ^###^P < 0.001

TEM analysis of the particle sizes of NPS, YQHXF-NPS, and MM/YQHXF-NPS revealed that all three nanospheres were approximately 100 nm in size, round in shape, and uniform in morphology, demonstrating good dispersion (Fig. 2A). Furthermore, mapping revealed that the nanoparticles contained a C/N/O/P elemental composition, with the P content increasing after cell membrane coating (Fig. 2B). Moreover, DLS data indicated that the average hydrodynamic size of NPS was 372.6 nm, with an average Polymer dispersity index (PDI) of 0.132. The average hydrodynamic size of YQHXF-NPS was 373.1 nm, with an average PDI of 0.296. After cell membrane coating, the average hydrodynamic size was 431 nm, with an average PDI of 0.152. The hydrodynamic size of NQHXF-NPS (MM/YQHXF-NPS) increased after macrophage membrane coating, while the PDI of the nanoparticles was relatively low, demonstrating good monodispersity (Fig. 3C). Notably, ZETA data showed that the surface potentials of NPS, YQHXF-NPS, and MM/YQHXF-NPS were − 0.802 mV, − 0.878 mV, and − 0.852 mV, indicating a weak negative charge on the nanoparticle surface. The encapsulation efficiency of YQHXF was 59.4%, and the drug loading rate was 5.61% (Figure S1C). Studies on the blood circulation and arterial targeting of YQHXF-NPs and MM/YQHXF-NPs in mice showed that MM/YQHXF-NPs had better targeting and blood retention capabilities (Figure S1A and B). One week of zeta potential monitoring demonstrated that the nanomaterial MM/YQHXF-NPs exhibited good stability in serum-containing medium (Figure S1D).

### Effects of MM/YQHXF-NPS on the transformation of A7r5 cells into foam cells

First, YQHXF effectively inhibited lipid droplet formation in the A7r5 model (Figure S1E and F). The cytotoxicity of 50, 100, and 150 μg/mL NPS, YQHXF-NPS, and MM/YQHXF-NPS against A7r5 cells was screened. As shown in Fig. 3A–C, these three nanomaterials showed no cytotoxicity, so a concentration of 150 μg/mL was selected for subsequent experiments. ox-LDL was used to construct a foam cell model and promote the transformation of A7r5 cells into foam cells. As shown in Fig. 3D, compared with the control group, the proliferation ability of the foam cell model constructed with ox-LDL was weakened. Compared with the model group, both YQHXF-NPS and MM/YQHXF-NPS promoted the proliferation of A7r5 cells. As shown in Fig. 3D, compared with the control group, the proliferation ability of the foam cell model constructed with ox-LDL was weakened. Compared with the model group, both YQHXF-NPS and MM/YQHXF-NPS promoted the proliferation of A7r5 cells. Oil red O staining was used to detect lipophagy by smooth muscle cells. Compared with the control group, a large amount of fat was detected in the model group. Compared with the model group, the amount of fat detected by YQHXF-NPS and MM/YQHXF-NPS was reduced (Fig. 3E and F). Furthermore, to elucidate the regulatory effects of the active ingredient Salvianolic acid B on the phenotypic switching and foam cell formation of vascular smooth muscle cells, this study employed an A7r5 foam cell formation model. It administered low (100 nM) and high (10 μM) doses of Salvianolic acid B as interventions. As shown in Figure S2, treatment with Salvianolic acid B reduced intracellular lipid accumulation, as visualized by Oil Red O staining. Mechanistically, Salvianolic acid B not only downregulated the expression of KLF4, a key protein involved in phenotypic switching that is elevated in the model group, but also inhibited the phosphorylation-mediated activation of the p65 protein (p-p65).

### MM/YQHXF-NPs regulate the alleviation of foamy phenotype of smooth muscle cells

**Fig. 4:**
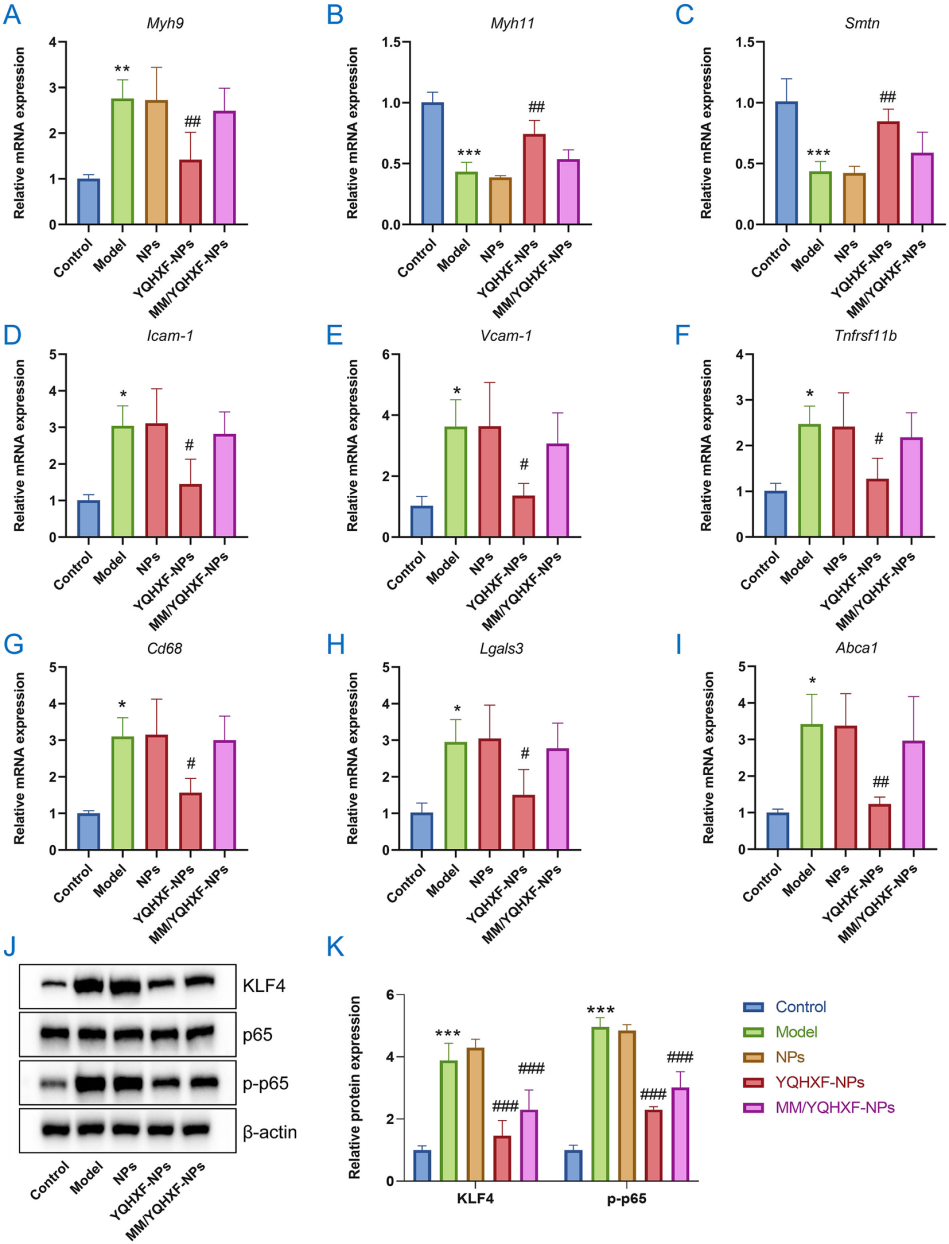
MM/YQHXF-NPs modulate the phenotype of rat smooth muscle cells. RT-qPCR analysis of the expression of contractile phenotype markers **A** *Myh9*, **B** *Myh11*, **C** *Smtn*; the transitional inflammatory markers **D** *Icam-1*, **E** *Vcam-1*, **F** *Tnfrsf11b*; and the macrophage phenotype markers **G** *Cd68*, **H** *Lgals3*, and (**I**) *Abca1* in aortic smooth muscle cells. (**J** and **K**) Representative protein bands and relative expression statistics for KLF4, p65, and p-p65. Data are presented as mean ± standard deviation, n = 3. Compared with the control group, *P < 0.05 and ***p < 0.001. Compared with the model group, ^#^P < 0.05, ^##^P < 0.01, and ^###^P < 0.001

As for *Myh9*, *Myh11*, and *Smtn*, compared with the control group, the model group showed increased *Myh9* expression, while *Myh11* and *Smtn* expression levels decreased. Compared with the model group, YQHXF-NPS and MM/YQHXF-NPS showed a trend of decreasing *Myh9* expression, while YQHXF-NPS inhibited the expression of *Myh11* and *Smtn* (Fig. 4A–C). As shown in Fig. 4D–F, compared with the control group, the expression of *Icam-1*, *Vcam-1*, and *Tnfrsf11b* increased in the model group. Compared with the model group, NPs did not affect the expression of *Icam-1*, *Vcam-1*, and *Tnfrsf11b*, while YQHXF-NPS and MM/YQHXF-NPS had a certain inhibitory effect on the expression of *Icam-1*, *Vcam-1*, and *Tnfrsf11b*. *Cd68*, *Lgals3*, and *Abca1* are markers of macrophages. Compared with the control group, the model group showed increased expression of *Cd68*, *Lgals3*, and *Abca1*. YQHXF-NPS, however, reduced the expression of these genes (Fig. 4G–I). Protein detection results showed that the expression of KLF4 and p-p65 was increased in the model group compared with the control group. Compared with the model group, both YQHXF-NPS and MM/YQHXF-NPS inhibited the expression of these two proteins (Fig. 4J and K).

### MM/YQHXF-NPs inhibit the transformation of A7r5 cells into foam cells by suppressing KLF4 and NF-κB signaling pathways

**Fig. 5:**
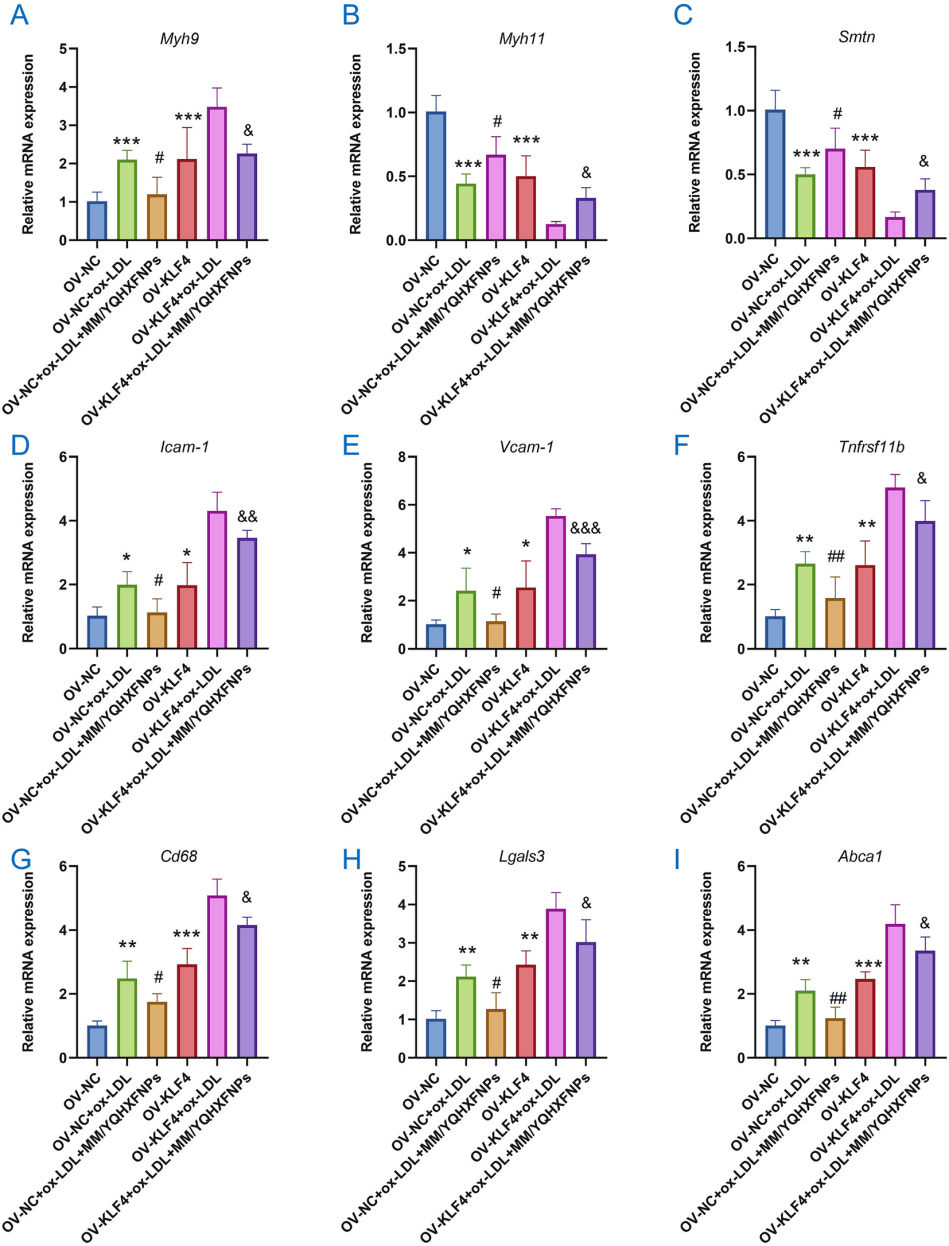
MM/YQHXF-NPs inhibit ox-LDL-induced foam cell formation in A7r5 cells. RT-qPCR analysis of the expression of contractile phenotype markers **A** *Myh9*, **B** *Myh11*, **C** *Smtn*; the transitional inflammatory markers **D** *Icam-1*, **E** *Vcam-1*, **F** *Tnfrsf11b*; and the macrophage phenotype markers **G** *Cd68*, **H** *Lgals3*, and **I** *Abca1* in aortic smooth muscle cells. Data are presented as mean ± standard deviation, n = 3. Compared with OV-NC, *P < 0.05, **P < 0.01, and ***P < 0.001. Compared with OV-NC + ox-LDL, ^#^P < 0.05 and ^##^P < 0.01. Compared with OV-KLF4 + ox-LDL, ^&^P < 0.05 and ^&&^P < 0.01

**Fig. 6:**
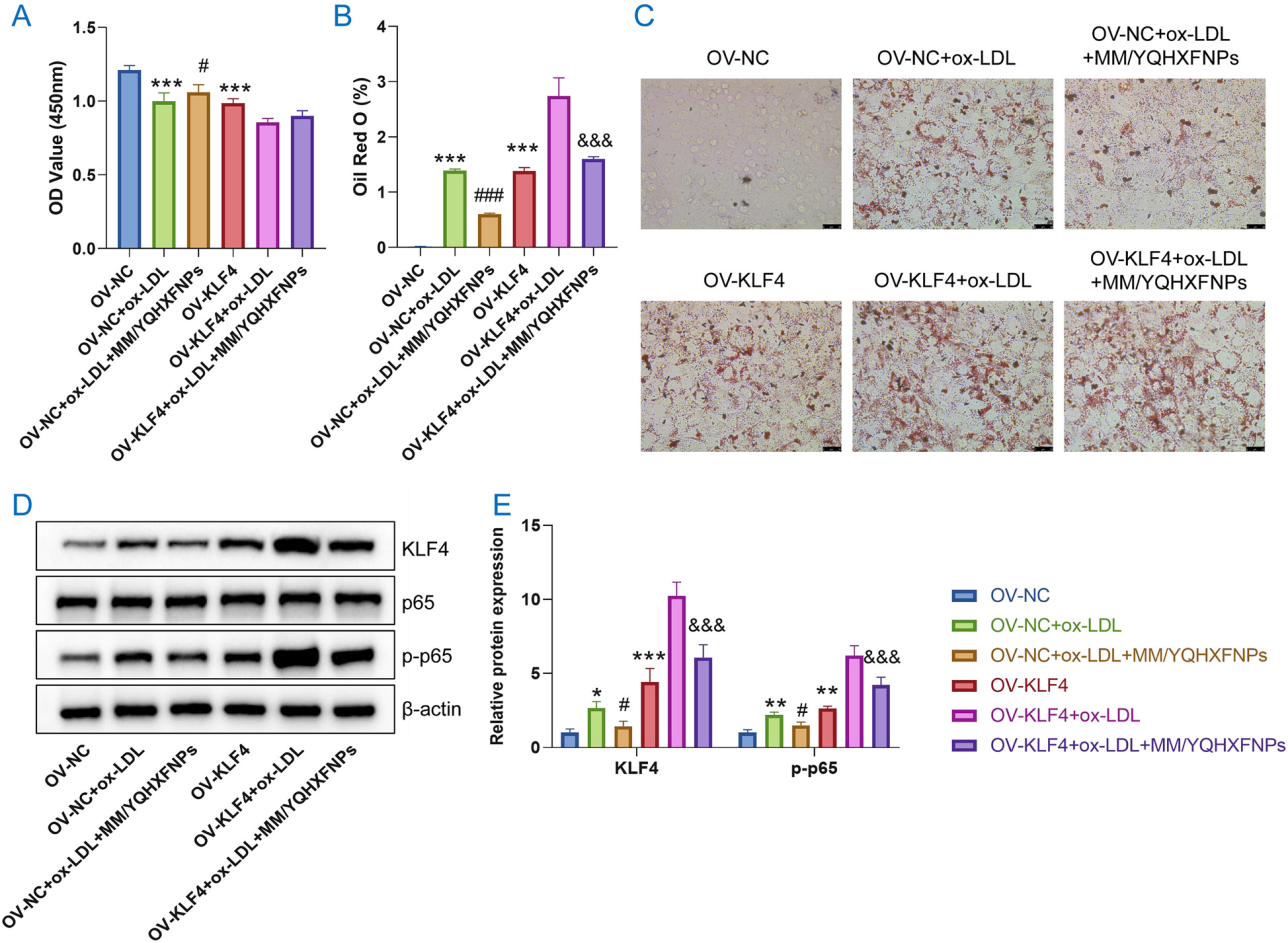
Effects of MM/YQHXF-NPs on A7r5 cell foaming via KLF4 and NF-κB signaling. **A** Cell proliferation assay using CCK-8. **B**, **C** Oil Red O staining results and representative images. **D**, **E** Representative protein bands and relative expression statistics for KLF4, p65, and p-p65. Data are presented as mean ± standard deviation, n = 3. *P < 0.05, **P < 0.01, and ***P < 0.001, Compared with OV-NC + ox-LDL, ^#^P < 0.05 and ^##^P < 0.01. Compared with OV-KLF4 + ox-LDL, ^&&^P < 0.01

Ox-LDL was used to induce A7r5 cell-to-foam cell transformation. As shown in Fig. 5A–I, ox-LDL and overexpression of KLF4 upregulated *Myh9* and inhibited the expression of *Icam-1*, *Vcam-1*, *Tnfrsf11b*, *Cd68*, *Lgals3*, and *Abca1*. Ox-LDL and OV-KLF4 had similar effects on A7r5 cells. Furthermore, MM/YQHXF-NPs inhibited the upregulation of *Myh9* induced by ox-LDL and OV-KLF4 and promoted the downregulation of *Icam-1*, *Vcam-1*, *Tnfrsf11b*, *Cd68*, *Lgals3*, and *Abca1* induced by ox-LDL and OV-KLF4. Furthermore, MM/YQHXF-NPs ameliorated the reduced proliferation of A7r5 cells induced by ox-LDL and OV-KLF4 (Fig. 6A). As shown in Fig. 6B and C, ox-LDL and OV-KLF4 increased the lipid staining area in A7r5 cells. Conversely, MM/YQHXF-NPs reduced the Oil Red O staining area. Regarding KLF4 and NF-κB proteins, ox-LDL and OV-KLF4 activated KLF4 expression and enhanced the phosphorylation of NF-κB p-p65. In addition, MM/YQHXF-NPs inhibited the activation of KLF4 and NF-κB p-p65 (Fig. 6D and E).

### MM/YQHXF-NPs alleviate AS in mice

**Fig. 7:**
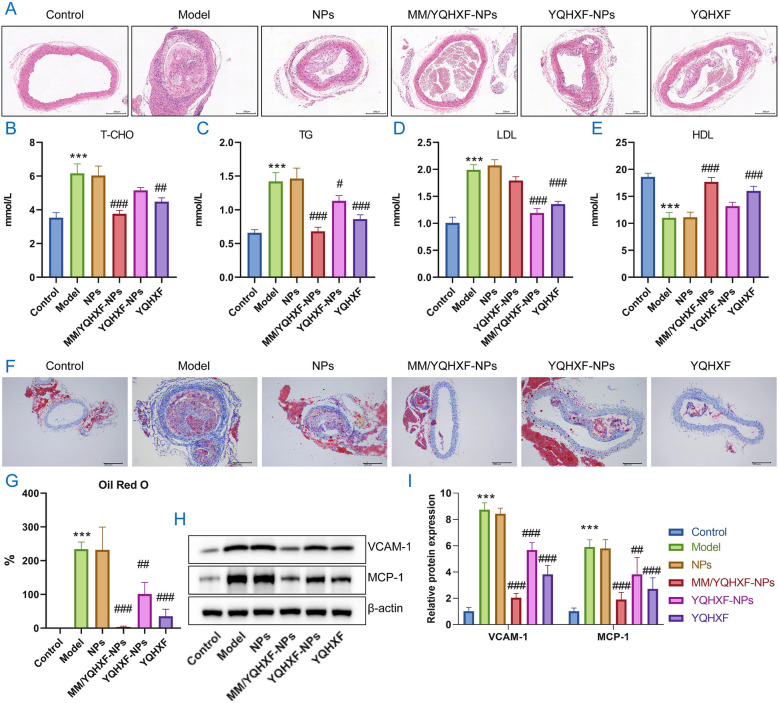
MM/YQHXFNPs alleviate AS in mice. **A** H&E staining of the aorta. **B**–**E** Serum TC, TG, LDL, and HDL levels in mice. **F**, **G** Oil Red O staining of the aorta and statistical results. **H**, **I** Representative images and relative expression of VCAM-1 and MCP-1. Data are presented as mean ± standard deviation, n = 6. Compared with the control group, ***p < 0.001. Compared with the model group, ^##^P < 0.01, and ^###^P < 0.001

Initially, H&E staining of liver tissue was performed for model evaluation and preliminary assessment of systemic safety (Figure S1G). H&E staining revealed that, compared with the control group, the model group exhibited pathological changes, including plaques, necrosis, and increased fibrous cap thickness. Pathological changes in the NPs group were similar to those in the model group. Compared with the model group, MM/NQHXF-NPS, YQHXF-NPS, and YQHXF all inhibited these pathological changes, with MM/NQHXF-NPS demonstrating the better therapeutic effect (Fig. 7A). Further analysis of serum TC, TG, LDL, and HDL revealed increased levels in the model group compared to the control group. These levels were nearly identical between the NPs and model groups. Compared to the model group, MM/NQHXF-NPS, YQHXF-NPS, and YQHXF all reduced serum TC, TG, LDL, and HDL levels in mice with AS, with MM/NQHXF-NPS demonstrating the better therapeutic effect (Fig. 7B–E). As shown in Fig. 7F and G, compared with the control group, the blood vessels of the model group mice were widely stained with Oil Red O. The Oil Red O staining area was reduced after MM/NQHXF-NPS, YQHXF-NPS, and YQHXF treatment compared with the model group. Additionally, protein analysis revealed that the expression of VCAM-1 and MCP-1 was increased in the model group compared to the control group. Compared with the model group, MM/NQHXF-NPS, YQHXF-NPS, and YQHXF inhibited the expression of these two proteins, with MM/NQHXF-NPS having a more significant effect (Fig. 7H and I).

### Effects of MM/YQHXF-NPs on atherosclerotic plaque stability

**Fig. 8:**
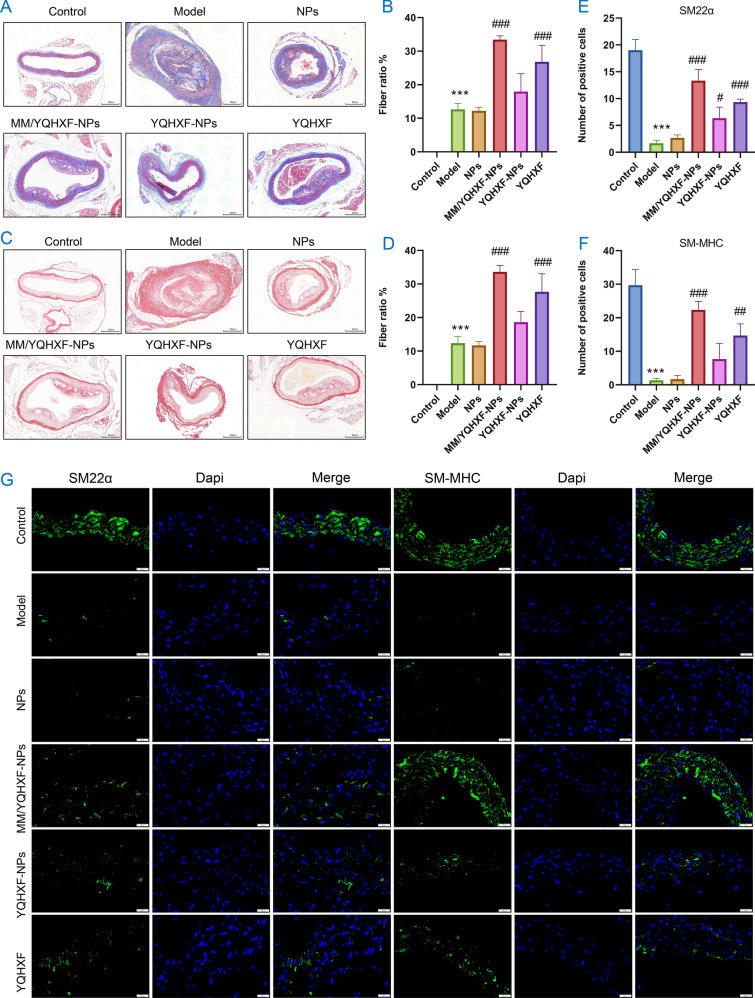
Fibrosis of plaques in aortic tissue. **A**, **B** Masson staining of the aorta. **C**, **D** Picrosirius red staining of the aorta. **E** Immunofluorescence analysis of SM22α. **F** Immunofluorescence analysis of SM-MHC. **G** Representative images of SM22α and SM-MHC immunofluorescence. Data are presented as mean ± standard deviation, n = 6. Compared with the control group, ***p < 0.001. Compared with the model group, ^#^P < 0.05, ^##^P < 0.01, and ^###^P < 0.001

As shown in Fig. 8A and B, the percentage of fiber area within aortic tissue plaque in the model group increased compared with the control group. While the percentage of fiber area in the NPs group remained similar to that in the model group, the percentage of fiber area in the MM/NQHXF-NPS, YQHXF-NPS, and YQHXF groups increased. Compared with the YQHXF group, the percentage of fiber area in the MM/YQHXF-NPs group increased. Consistent with the results of Masson staining, Sirius red staining revealed an increase in the percentage of fibrotic plaque area within the model group compared with the control group. Compared with the model group, the degree of fibrosis was increased after treatment with MM/NQHXF-NPS, YQHXF-NPS, and YQHXF (Fig. 8C and D). Immunofluorescence staining of SM22α and SM-MHC expression in the aorta (Fig. 8E–G). Compared with the control group, the expression of SM22α and SM-MHC in the model group was reduced. The expression of SM22α and SM-MHC in the model group and the NPs group was consistent. Compared with the model group, the expression of SM22α and SM-MHC in the MM/NQHXF-NPS, YQHXF-NPS, and YQHXF groups increased, with MM/NQHXF-NPS having a more significant effect.

### Effects of MM/YQHXF-NPs on regulating the phenotype of mouse smooth muscle cells

**Fig. 9:**
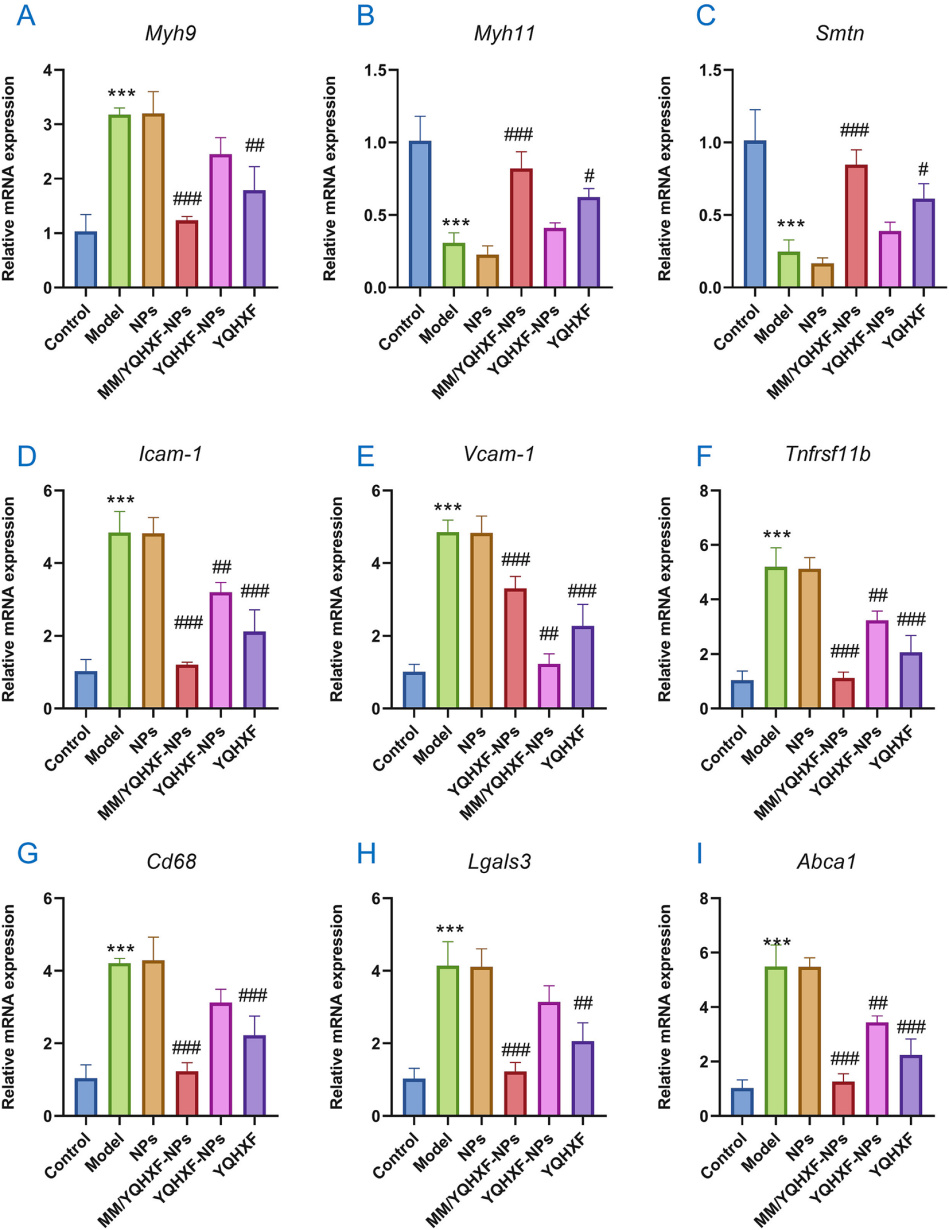
MM/YQHXF-NPs inhibit the transformation of SMCs into foam cells. RT-qPCR analysis of the expression of contractile phenotype markers **A** *Myh9*, **B** *Myh11*, **C** *Smtn*; the transitional inflammatory markers **D** *Icam-1*, **E** *Vcam-1*, **F** *Tnfrsf11b*; and the macrophage phenotype markers **G** *Cd68*, **H** *Lgals3*, and **I** *Abca1* in mouse aortic smooth muscle cells. Data are presented as mean ± standard deviation, n = 6. Compared with the control group, ***p < 0.001. Compared with the model group, ^#^P < 0.05, ^##^P < 0.01, and ^###^P < 0.001

To further verify the effect of MM/YQHXF-NPs on the phenotype of A7r5 cells in cell experiments, RT-qPCR was used to detect the changes of *Myh9*, *Myh11*, *Smtn*, *Icam-1*, *Vcam-1*, *Tnfrsf11b*, *Cd68*, *Lgals3*, and *Abca1* in mouse arterial tissue. As shown in Fig. 9A–C, compared with the control group, the expression of *Myh9* increased in the model group, while the expressions of *Myh11* and *Smtn* decreased. Compared with the model group, NPs did not affect *Myh9*, *Myh11*, and *Smtn*. MM/NQHXF-NPS, YQHXF-NPS, and YQHXF inhibited the expression of *Myh9* and promoted the expression of *Myh11* and *Smtn*. As for *Icam-1*, *Vcam-1*, and *Tnfrsf11b*, the expression of these three genes increased in the model group compared with the control group. Compared with the model group, both YQHXF and YQHXF-prepared nanomaterials inhibited the expression of these three genes, with MM/NQHXF-NPS having the best inhibitory effect (Fig. 9D–F). In addition, compared with the control group, the expressions of *Cd68*, *Lgals3*, and *Abca1* were increased in the model group, while MM/NQHXF-NPS, YQHXF-NPS, and YQHXF inhibited the expression of these macrophage markers (Fig. 9G–I).

### Effects of MM/YQHXF-NPs on KLF4 and NF-κB signaling pathways

**Fig. 10:**
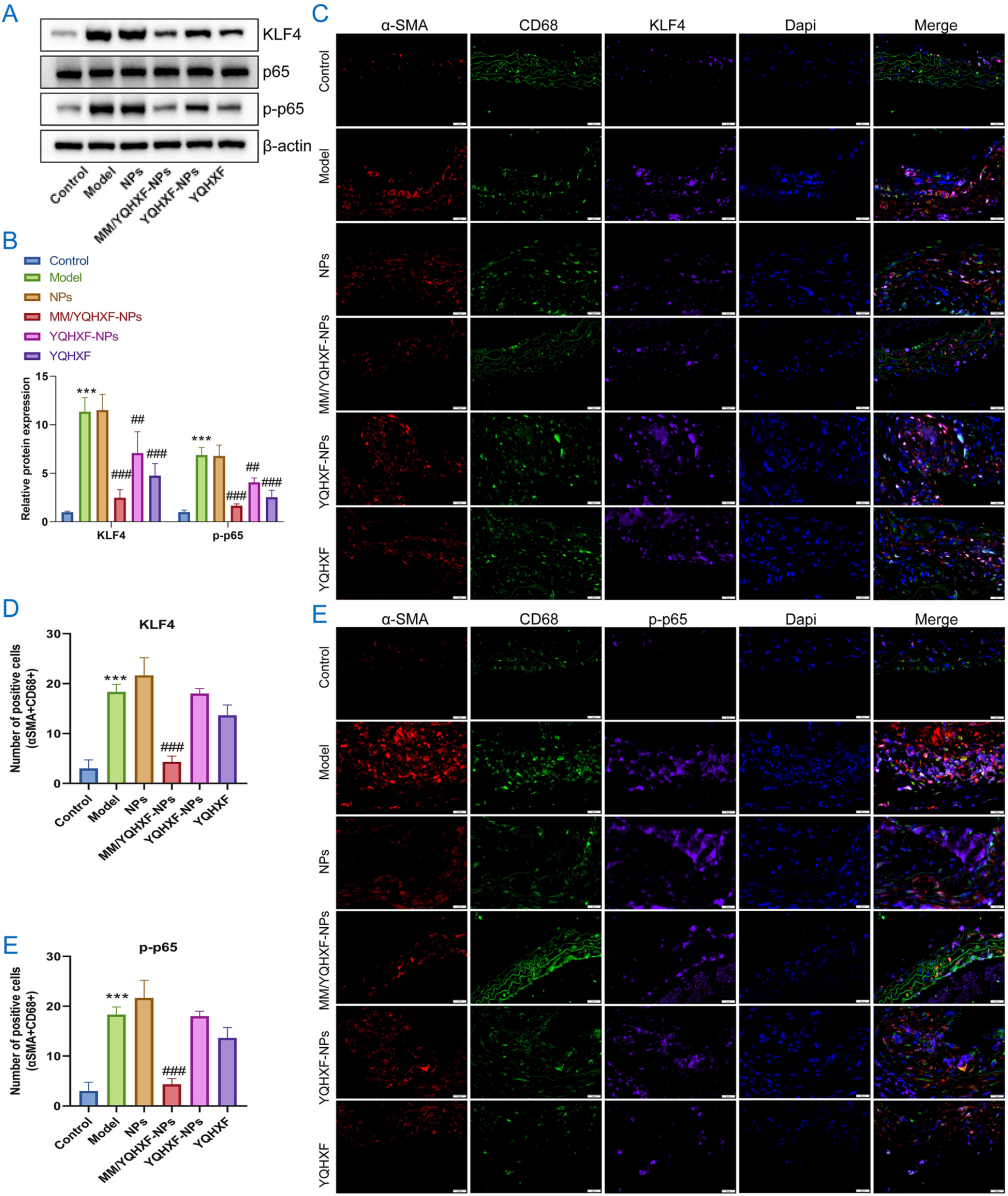
MM/YQHXF-NPs inhibit KLF4 and NF-κB signaling pathway activation in mice with AS. **A**, **B** Representative protein bands and relative expression statistics for KLF4, p65, and p-p65. **C**–**E** Representative images of triple immunofluorescence staining of KLF4/CD68/α-SMA and p-p65/CD68/α-SMA in mouse aortas, and statistical analysis of triple-stained cells. Data are presented as mean ± standard deviation, n = 6. Compared with the control group, ***p < 0.001. Compared with the model group, ^##^P < 0.01, and ^###^P < 0.001

WB assayed the expression levels of KLF4, p65, and p-p65 in mouse aortas. Compared with the control group, KLF4 and p-p65 expression increased in the model group. Compared with the model group, MM/NQHXF-NPS, YQHXF-NPS, and YQHXF all inhibited the expression of these two proteins. Furthermore, the expression levels of KLF4 and p-p65 in the MM/NQHXF-NPS group were lower than those in the YQHXF-NPS and YQHXF groups (Fig. 10A and B). Delving into the realm of immunofluorescence staining, we found that the model group showed increased expression of KLF4 and p-p65 compared to the control group. The expression of these two proteins was consistent between the model and NPs groups. Compared to the model group, MM/NQHXF-NPS inhibited the expression of KLF4 and p-p65 (Fig. 10C–E).

## Discussion

Targeting the characteristics of AS lesions, a macrophage membrane-coated biomimetic nanodrug, MM/YQHXF-NPs, was prepared by combining macrophage membranes with an "artificial" polymer nanodrug delivery system design strategy. This study systematically investigated the prepared MM/YQHXF-NPs, focusing on characterizing their biomimetic nanodrug properties, conducting in vitro cell experiments, and evaluating their in vivo therapeutic efficacy and safety. Overall, the MM/YQHXF-NPs biomimetic nanodrugs prepared in this study exhibit excellent nanodrug properties, enabling long-term circulation in the body, achieving efficient targeted aggregation and drug delivery at AS lesions, effectively inhibiting the development of AS plaques, and exhibiting a good safety profile.

Statins are commonly used clinical medications for the treatment of AS. Statins primarily act by competitively inhibiting hydroxymethylglutaryl-coenzyme A reductase, thereby blocking hepatic cholesterol synthesis; their core mechanism lies in "lipid-lowering" effects [37]. TCM compounds contain a diverse array of active ingredients that can simultaneously intervene in multiple pathological processes associated with AS [28, 30–32]. Beyond regulating lipid metabolism, their advantages are further demonstrated by their anti-inflammatory and antioxidant properties, as well as their ability to enhance vascular endothelial function. According to TCM theory, AS is classified under the categories of "true heart pain," "chest Bi," and "vessel impediment." In contemporary medical terminology, it is associated with coronary heart disease (CHD) [38]. CHD is characterized by chest pain in the classical medical texts, the Yellow Emperor's Inner Canon (Huang Di Nei Jing) and the Synopsis of the Golden Chamber (Jin Kui Yao Lüe Fang Lun) [39]. TCM have demonstrated promising efficacy in the prevention and treatment of AS, and research on the effectiveness and mechanisms of action of these Traditional Chinese herbal medicines against AS is increasing [40]. With its multi-pathway and multi-target approach, TCM offers advantages in the diagnosis and treatment of AS [41]. The YQHXF contains hong shen, cang zhu, dan shen, chuan xiong, san qi, herbs commonly used to treat cardiovascular disease and AS [24–27, 42]. Among these, the active ingredient Salvianolic acid B has been demonstrated to possess anti-atherosclerotic effects [31]. Salvianolic acid B inhibits NF-κB activity, thereby suppressing the progression of inflammation [32]. Although most Traditional Chinese herbal medicines are considered relatively safe, their complex composition carries the potential for adverse reactions [43]. Furthermore, some herbal components may adversely affect organs such as the liver and kidneys, and long-term use may increase liver and kidney burden [43]. Therefore, there is an urgent need to combine TCM preparation technology with modern science and technology to improve its modernization level. PLGA is an FDA-approved biodegradable polymer that hydrolyzes in vivo into non-toxic lactic acid and glycolic acid, thereby ensuring excellent long-term safety [44]. Preliminary safety data for the formulation investigated in this study indicate that it exhibits no significant systemic toxicity.

Nanotechnology can address the problem of TCM extracts, which often destroy their bioactive ingredients, leading to low absorption and limited efficacy [45]. Nanomaterialization of TCMs can significantly alter their physical and biological properties. Nanotechnology can be used to transform TCMs into nanocapsules or nanopowders for injection. It can also process poorly soluble and insoluble drugs into nanoparticles, improving absorption and bioavailability, enhancing efficacy, and reducing toxic side effects [46]. Wang [47] found that, compared to drug-loaded formulations, macrophage membrane-coated blank carriers (MM-NPs) lacked potent pharmacological activity in inhibiting vascular inflammation or plaque progression. Nanoparticles wrapped in macrophage membranes have good anti-immune clearance capabilities and can circulate in the blood for a long time [48]. Particle size analysis, potentiometric analysis, TEM testing, and drug loading and encapsulation efficiency measurements of MM/YQHXF-NPs in this study demonstrate that these nano-TCMs are nanoscale, uniform in size, and well-dispersed, achieving efficient drug encapsulation. We have successfully constructed macrophage membrane-coated YQHXF-NPs. Although similar preliminary studies suggest that macrophage membranes primarily function as a targeting shell and exert minimal independent therapeutic effects, their intrinsic biological activity constitutes a complex factor that warrants further investigation.

Our in vitro studies confirmed that NPS, YQHXF-NPS, and MM/YQHXF-NPS had no significant cytotoxicity against A7r5 smooth muscle cells. Vascular SMCs exhibit robust phenotypic plasticity [49]. In response to pathological stimuli, such as growth factors and inflammatory factors, SMCs can transform a contractile phenotype to a secretory or inflammatory phenotype [49]. Low-density lipoprotein (LDL) and its modified form, ox-LDL, play a major role in the development of AS and foam cell formation [50]. Although the majority of these cells have been shown to originate from macrophages, SMCs also generate a significant number of foam cells [50]. Similar to previous studies, we used ox-LDL stimulation of A7r5 cells, which resulted in enhanced Oil Red O staining and the acquisition of a foam cell phenotype. Furthermore, during atherosclerotic plaque formation, SMCs transition from a contractile to migratory and phagocytic phenotype [51]. Previous studies have shown that *Myh9* expression is significantly upregulated in human atherosclerotic samples and various animal models of AS [52]. Furthermore, the *Myh11* protein can generate contractile force by interacting with actin [53]. *Smtn* vascular SMCs maintain their contractile capacity to maintain normal vascular tone and caliber [54]. In this study, both MM/YQHXF-NPs and YQHXF-NPs inhibited the phagocytic phenotype of A7r5 cells after ox-LDL exposure.

In the in vivo experiment, we selected *ApoE*^−/−^ mice fed a high-fat diet as the research subjects. *ApoE*^−/−^ mice are currently the most widely used mice for AS research [55]. Under a normal diet, the TC and LDL-C levels of *ApoE*^−/−^ mice are several times those of wild-type mice [56]. However, *ApoE*^−/−^ mice fed a high-fat diet showed the pathological manifestations of AS in the middle and late stages, accompanied by the deposition of lipids in the aorta [56]. In this study, *ApoE*^−/−^ mice fed a high-fat diet exhibited mid-to-late stage pathological manifestations of AS. MM/YQHXF-NPs significantly alleviated AS in these high-fat-fed *ApoE*^−/−^ mice. Based on histopathological findings, lipid profiles, and protein expression, MM/YQHXF-NPs, YQHXF-NPs, and YQHXF all inhibited pathological changes, including plaque formation, necrosis, and fibrous cap thickening. MM/YQHXF-NPs significantly inhibited the deposition of lipid plaques in the aortas of AS mice and reduced serum levels of TC, TG, LDL, and HDL.

Inflammation plays a crucial role in the development and progression of AS [57]. Inflammatory cell infiltration and cytokine release affect the formation and stability of AS plaques [57]. The activation of the inflammatory cascade in vascular endothelial cells stimulates the *Vcam-1*, *Icam-1*, and MCP-1, promoting the recruitment and deposition of circulating monocytes in the intimal inflammatory sites, and differentiation into macrophages and foam cells, which secrete a large number of pro-inflammatory factors to aggravate atherosclerotic lesions [58]. In addition, TNF-α induces a pro-inflammatory phenotype in vascular SMCs through a platelet-derived growth factor receptor β-dependent pathway [59]. *Tnfrsf11b* is a member of the TNF receptor superfamily and has been reported to predict AS [60]. Our in vitro and in vivo studies have shown that MM/YQHXF-NPs can inhibit the gene expression of pro-inflammatory factors in ox-LDL-induced A7r5 cells and high-fat-induced AS mice.

Macrophages can ingest lipid deposits in the vessel wall and increase inflammatory responses [61]. Macrophage accumulation and activation in AS lesions are positively correlated with lesion severity [62]. Zhai et al. [63] reported that macrophage transdifferentiation of vascular SMCs can activate the TLR4 and STING-SOCS1 signaling pathways in other cells within the plaque, accelerating the pathogenic transdifferentiation of vascular SMCs and promoting plaque progression and instability. Our studies have found that MM/YQHXF-NPs inhibit the ox-LDL-induced transformation of A7r5 cells into a macrophage phenotype. Furthermore, in vivo experiments demonstrated that MM/YQHXF-NPs, YQHXF-NPs, and YQHXF inhibited the expression of *Cd68* [64], *Lgals3* [65], and *Abca1* [66] in AS mice, leading to a decrease in macrophage markers. Therefore, constructing nanoparticle systems that target macrophages and foam cells in AS plaques is an effective approach for the treatment of AS.

AS is an important pathological basis for cardiovascular disease, and the stability of its plaques directly affects the progression and prognosis of the disease [67]. A smaller lipid core and a fibrous cap with intact structure and uniform thickness characterize stable plaques [68]. In contrast, once the fibrous cap becomes thinner and the elastic fibers and collagen fibers decrease, it is often a precursor to plaque rupture [69]. Additionally, the formation and maintenance of the plaque's fibrous cap primarily depend on the collagen produced by vascular smooth muscle cells. The thicker the fibrous cap and the higher the collagen content, the more stable the plaque and the lower the risk of rupture [70]. In this study, MM/YQHXF-NPs induced SM22α [71] and SM-MHC [72] in the blood vessels of AS mice, indicating the restoration of vascular stability and function. SM22α is a key cytoskeletal protein required for maintaining the contractile phenotype of VSMCs and is a highly sensitive and specific marker for identifying phenotypic transformation of VSMCs [72]. Our tissue staining also demonstrated that MM/YQHXF-NPs protected collagen, suggesting increased plaque stability.

KLF4 is a transcription factor containing three zinc finger structures [73]. It can participate in different cell signaling networks, regulate the transcriptional activation or inhibition of target genes, and has bidirectional regulatory functions such as cell proliferation and differentiation, oncogenes and tumor suppressor genes, and pro-inflammatory and anti-inflammatory functions [73]. Importantly, Klf4 knockout in SMCs in AS resulted in a decrease in the number of SMC-derived macrophage-like cells, a significant reduction in lesion size, and an improvement in multiple indicators of plaque stability, including an increase in fibrous cap thickness [14, 74]. Regarding NF-κB, intermittent hypoxia induces an inflammatory response in AS by activating the TLR4/NF-κB signaling pathway [75]. *ApoE*^−/−^ mice lacking TLR4 or MyD88 have significantly reduced AS plaques and more stable plaques [76]. Some studies [20, 21] reported that reducing the expression of KLF4 can inhibit the activation of NF-κB p65 and protect against cardiovascular damage. Our in vitro experiments demonstrated that MM/YQHXF-NPs can inhibit the phosphorylation of activated KLF4 and NF-κB p65. Overexpression of KLF4 in A7r5 cells enhanced the phosphorylation of NF-κB p65, and A7r5 cells switched from a contractile phenotype to a migratory and phagocytic phenotype. In vivo experiments also demonstrated that MM/YQHXF-NPs, YQHXF-NPs, and YQHXF all inhibited KLF4 and p-p65 protein expression in mouse arteries, with the MM/YQHXF-NPs group showing the most significant inhibitory effect. MM/YQHXF-NPs also inhibited KLF4 and p-p65 expression in a-SMA- and CD68-positive cells, demonstrating the inhibitory effect of KLF4 and p-p65 signaling pathways on smooth muscle cell phenotypic transformation.

## Conclusions

This study prepared a biomimetic nanopharmaceutical, MM/YQHXF-NPs, with superior nanoscale properties. Through macrophage membrane coating and a synergistic design of an "artificial" polymer nanopharmaceutical delivery system, this drug achieved long-term in vivo circulation and efficient targeting of atherosclerotic (AS) lesions. MM/YQHXF-NPs effectively blocked the activation of proinflammatory signaling pathways by inhibiting the phosphorylation of the key transcription factor KLF4 and its downstream NF-κB p65, thereby suppressing the abnormal phenotypic transformation of smooth muscle cells. However, achieving large-scale, standardized production and establishing a rigorous quality control system to ensure batch-to-batch consistency and stability remain challenges.

## Supplementary Information

Supplementary material 1.

Supplementary material 2.

## Data Availability

The datasets used or analyzed during the current study are available from the corresponding author on reasonable request.

## Abbreviations

KLF4: Krüppel-like factor 4
SMCs: smooth muscle cells
DLS: Dynamic light scattering
AS: Atherosclerosis
TEM: transmission electron microscopy
VSMCs: Vascular smooth muscle cells
ECM: extracellular matrix
