# Supplementary material for: Macrophage membrane-functionalized biomimetic Yiqi Huoxue formula nanoparticles improve atherosclerosis by regulating smooth muscle cell phenotypic transition via the KLF4/NF-κB pathway
Source: Chin Med. 2026 Jul 27;21:203. doi: 10.1186/s13020-026-01477-y (PMC13404646; doi:10.1186/s13020-026-01477-y)
Supplement: Supplementary file 1 — Supplementary material 1. [file 13020_2026_1477_MOESM1_ESM.doc]

***In vivo* AS targeting studies**

Grouping: YQHXF-NPs, MM/YQHXFNPs. 6 *ApoE*-/- mice were randomly divided into 2 groups of 3 after a high-fat diet for 6 or 8 weeks. Each group received a tail vein injection of 200 μL of a 2 mg/mL MM/YQHXFNPs solution, while the other group received an equal amount of YQHXFNPs. Twenty-four hours after injection, mice in each group were anesthetized and sacrificed. The mice were perfused with heparinized PBS. The artery was excised from the aortic root to the iliac bifurcation. Periaroflavones were carefully removed with forceps, and residual blood within the arterial lumen was washed with PBS to prevent nonspecific signal interference. The excised arteries were placed in a small animal imaging system and imaged using 640 nm excitation light. MM/YQHXFNPs detected a stronger signal than YQHXF-NPs (Figure S1A).

***In vivo* long-term circulation assay**

Groups: YQHXF-NPs, MM/YQHXF-NPs. Methods: Six 8-week-old C57BL/6 mice were randomly divided into two groups, with three mice in each group. Each group received a 200 μL tail vein injection of a 2 mg/mL YQHX-FNPs or MM/YQHXF-NPs nanoparticle solution. 30 μL of blood was collected from the tail of the mice 1 min, 1 h, 6 h, 12 h, 24 h, and 48 h after nanoparticle injection. The collected blood was immediately diluted with 30 μL of 2 mM EDTA-2K PBS. After all samples were collected, they were plated into 96-well plates, and fluorescence intensity was measured using a fluorescence microplate reader. MM/YQHXF-NPs exhibited stronger fluorescence in the mouse blood, indicating improved blood retention (Figure S1B).

**Zeta Potential Measurement**

MM/YQHXF-NPS was mixed with DMEM containing 10% serum. At designated time points (Days 1, 3, 5, and 7), 1 mL aliquots of the sample solution were withdrawn from the culture medium; an excess of acetonitrile was added to remove components of the medium, followed by centrifugation. The Zeta potential was then measured (Figure S1D).

**Preparation of Drug-Containing Serum**

Animals were administered the drug daily via oral gavage for three consecutive days at the dosage specified in the manuscript. Blood samples were collected one hour after the final administration, and the serum from two mice was pooled together. The pooled serum was then incubated in a 56°C constant-temperature water bath for 30 minutes to inactivate antibodies, followed by sterilization via filtration through a 0.22 μm microfiltration membrane; the prepared sample was then delivered to the cell culture facility. For the negative control serum, no drug was administered via oral gavage; instead, serum was collected directly, with all other procedures remaining identical. The obtained serum was utilized for the experiments presented in Figures S1E and S1F. The experimental results demonstrated that YQHXF effectively inhibited lipid droplet formation in the A7r5 foam cell model.

**Liver H&E Staining**

In the Model and NPs groups, hepatocytes were observed to contain intracytoplasmic, unstained, spherical lipid droplets of varying sizes, with small droplets predominating (Figure S1G). No significant changes were observed in the other groups.

Figure S1. Studies on the blood circulation, arterial targeting, and biological activity of YQHXF-NPs and MM/YQHXF-NPs. (A) Study of YQHXF-NPs and MM/YQHXF-NPs in mouse blood circulation and arterial targeting. (B) Fluorescence imaging of arteries 24 hours after tail vein injection of YQHXF-NPs and MM/YQHXF-NPs. Fluorescence intensity in the blood of mice at different time points after YQHXF-NPs and MM/YQHXF-NPs injection. (C) Table characterizing drug-loading properties: listing the encapsulation efficiency (EE%) and drug loading capacity (DL%) of the nanoparticles. (D) Zeta potential stability test: changes in the Zeta potential of MM/YQHXF-NPs incubated in DMEM medium containing 10% serum over a period of 7 days, reflecting the stability of the nanoparticles in a physiological environment. (E) and (F) Quantification and staining of lipid droplets in a foam cell model. (G) H&E-stained liver sections. Compared with the control group, ***p < 0.001. Compared with the model group, ###p < 0.001.


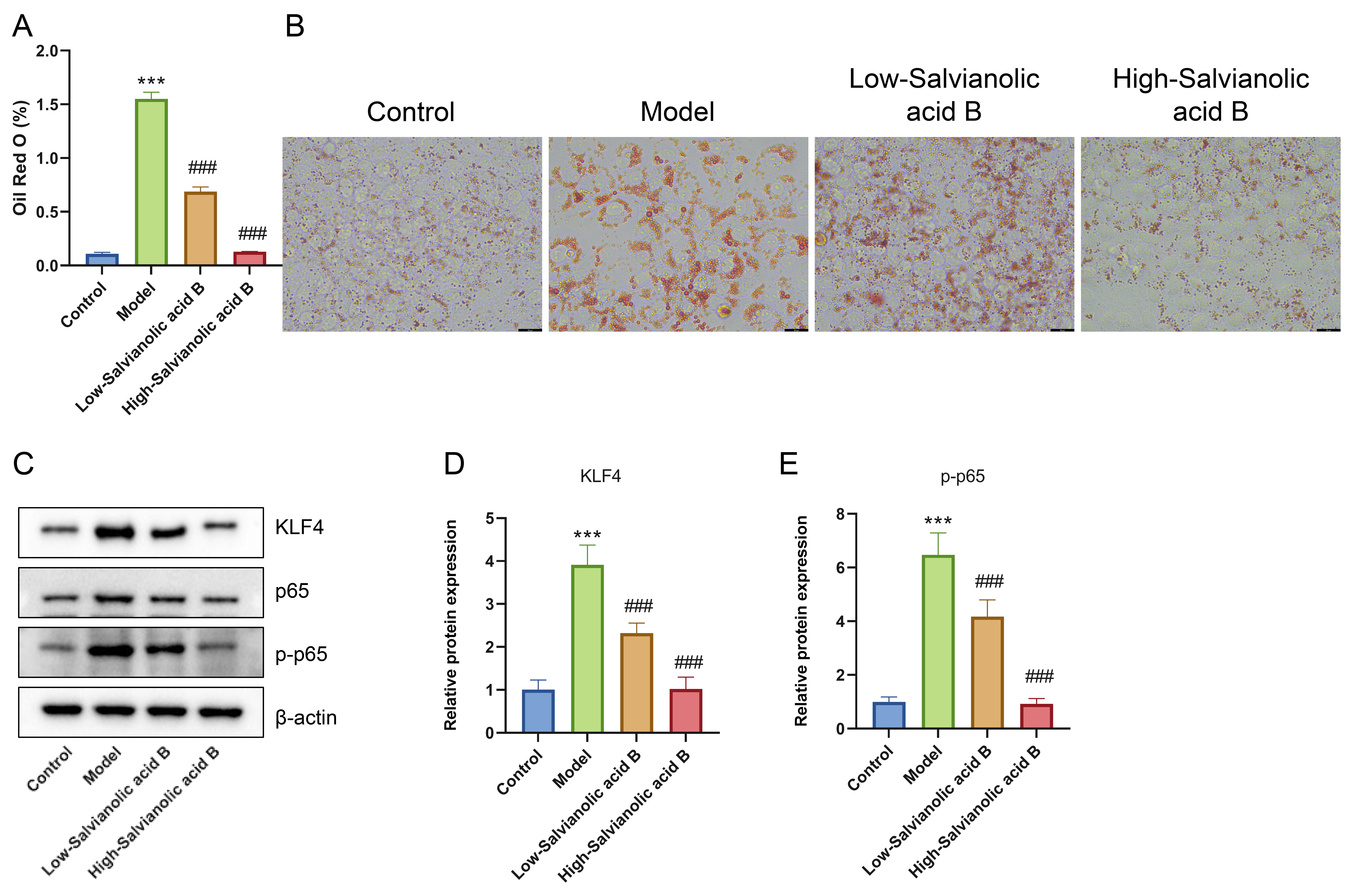


Figure S2. Effects of Salvianolic acid B on foam cell formation and the KLF4/p-p65 signaling pathway in A7r5 smooth muscle cells. (A) Quantitative analysis of Oil Red O staining in A7r5 cells across groups. (B) Microscopic images of Oil Red O staining in A7r5 cells across groups. (C) Western blot bands showing the expression of KLF4, p65, and p-p65 proteins in cells across groups. (D) Relative expression levels of KLF4 protein in cells across groups. (E) Relative expression levels of p-p65 protein in cells across groups. Compared with the control group, ***p < 0.001. Compared with the model group, ###p < 0.001.
